# Experimental realization of one dimensional helium

**DOI:** 10.1038/s41467-022-30752-3

**Published:** 2022-06-07

**Authors:** Adrian Del Maestro, Nathan S. Nichols, Timothy R. Prisk, Garfield Warren, Paul E. Sokol

**Affiliations:** 1 Department of Physics and Astronomy, University of Tennessee, Knoxville, TN 37996 USA; 2 Min H. Kao Department of Electrical Engineering and Computer Science, University of Tennessee, Knoxville, TN 37996 USA; 3 Institute for Advanced Materials and Manufacturing, University of Tennessee, Knoxville, TN 37996 USA; 4 Data Science and Learning Division, Argonne National Laboratory, Argonne, IL 60439 USA; 5 Division of Chemistry and Chemical Engineering, California Institute of Technology, Pasadena, CA 91125 USA; 6 Department of Physics, Indiana University, Bloomington, IN 47408 USA

**Keywords:** Quantum fluids and solids, Structural properties, Structure of solids and liquids, Nonlinear phenomena

## Abstract

As the spatial dimension is lowered, locally stabilizing interactions are reduced, leading to the emergence of strongly fluctuating phases of matter without classical analogues. Here we report on the experimental observation of a one dimensional quantum liquid of ^4^He using nanoengineering by confining it within a porous material preplated with a noble gas to enhance dimensional reduction. The resulting excitations of the confined ^4^He are qualitatively different than bulk superfluid helium, and can be analyzed in terms of a mobile impurity allowing for the characterization of the emergent quantum liquid beyond the Luttinger liquid paradigm. The low dimensional helium system offers the possibility of tuning via pressure—from weakly interacting, all the way to the super Tonks-Girardeau gas of strongly interacting hard-core particles.

## Abstract (web summary)

Helium isotopes are interesting platforms for testing the quantum properties of fluids. Here the authors demonstrate quantum one-dimensional behaviour of helium (4He) confined in nanopores by using neutron scattering.

## Introduction

The helium isotopes ^3^He and ^4^He have long served as model systems for precision tests of theories of strongly interacting quantum matter and phase transitions for bosons, fermions, and mixed statistics systems. This has been spectacularly successful in two^1^ and three dimensions^2^, where the dynamics can be understood in terms of quasi-particles which retain the original nature of the system through renormalized properties. In one spatial dimension (1D), the fundamental excitations of a quantum liquid of helium will be collective in nature, and at long wavelengths and low energies, it should be described via the linear hydrodynamics of Tomonaga-Luttinger liquid (TLL) theory^3–6^. Within this picture, the distinction between bosons and fermions begins to break down and access to both bosonic and fermionic isotopes makes 1D helium an exciting system to explore. This has motivated a number of experimental^7–12^ and theoretical^13–19^ studies in quasi-1D confinement, providing tantalizing evidence of low-dimensional or TLL behavior. However, as helium is a neutral quantum liquid, the route to 1D requires physical confinement in two out of three dimensions at the level of a single nanometer—the scale of the superfluid coherence length at low temperature—a difficult feat for real devices.

In this work, we introduce a nanoengineered confining environment, and report elastic and inelastic neutron scattering measurements of helium. By analyzing the observed excitation spectrum within the context of a non-linear Luttinger liquid we demonstrate the existence of generalized quantum hydrodynamics in one dimensional bosonic ^4^He.

## Results

### Confined helium

**Fig. 1:**
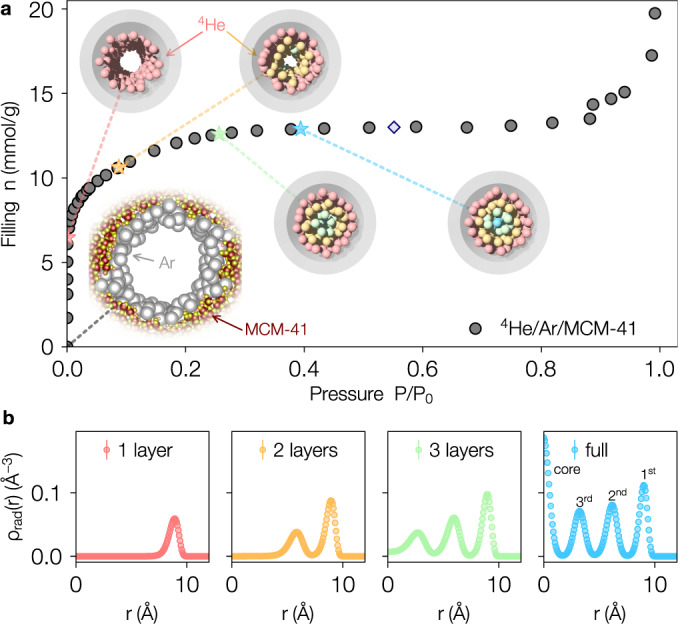
Adsorption and structure inside nanopores. **a** Dark gray circles illustrate the adsorption behavior of ^4^He at 4.2 K into MCM-41 pre-plated with a monolayer of Ar gas as the pressure is increased. Here *P*_0_ is the bulk equilibrium vapor pressure of ^4^He. The colored stars indicate the fillings where completion of ^4^He layers occurs with the call-out inset images showing quantum Monte Carlo configurations of a cross-section of MCM-41 with an equilibrated Ar layer (light gray spheres) at *P*/*P*_0_ = 0, and the developing layers of ^4^He (1 layer to 3 layers plus central core) as the pressure is increased. Here the Ar is represented as a cylindrical shell for clarity. The light purple diamond indicates the filling at which experimental inelastic neutron scattering measurements were performed at *Q*_in_ = 4.0 Å^−1^ corresponding to completely filled pores. **b** Quantum Monte Carlo results with binned stochastic errorbars for the radial number density of atoms *ρ*_rad_(*r*) inside nanopores at *T* = 1.6 K where the scattering experiments were performed. Colors correspond to the starred filling fractions in **a**. As the pressure is increased, the ^4^He atoms form a series of concentric layers, with the density of the outer layers also increasing.

Our confinement platform consists of ^4^He adsorbed inside MCM-41, a mesoporous material with a hierarchical structure consisting of cylindrical pores arranged in a hexagonal lattice, pre-plated with a single monolayer of argon^18^. The MCM-41 sample had an as-synthesized pore diameter of 3.0 ± 0.3 nm as determined by N_2_ isotherms, known to be too large to yield 1D confinement^20^. The effective pore diameter was reduced to 2 nm through a pre-treatment step where Ar gas was added at 90 K. The resulting Ar/MCM-41 confining environment was characterized via a ^4^He adsorption isotherm at *T* = 4.2 K combined with molecular dynamics and quantum Monte Carlo simulations as shown in Fig. 1 (for sample preparation and simulation details see the Methods section). The results demonstrate that the helium atoms are initially strongly bound to the Ar-plated pore walls at low fillings, and completely fill the pores at 13 mmol g^−1^. At intermediate filling, nested cylindrical layers of helium are formed and particle exchanges between them are suppressed.

### Static structure inside nanopores

Understanding this microscopic structure will be crucial to interpret excitations inside the pores as measured by both elastic and inelastic neutron scattering. It is important to note that the elastic scattering with zero energy transfer *E* = 0 at wavevector *Q*, *S*(*Q*, 0), is distinct from the static structure factor $S(Q)=\int\nolimits_{-\infty }^{\infty }S(Q,E)\,dE$ which averages the collective motion of the system over all time scales. The elastic scattering, in contrast, probes the static behavior of the system. For example, while bulk liquid helium exhibits a well defined *S*(*Q*) reflecting the dynamic correlations in the liquid^2^, the elastic scattering is identically zero. Helium confined in various porous media^11^ does exhibit elastic scattering due to the presence of solid layers strongly adsorbed on the pore boundaries (see Fig. 1b). However, in previously studied pores with large radii that are not yet in the quasi-1D regime, dense liquid layers and a bulk-like liquid in the center of the pore do not contribute to the elastic scattering.

**Fig. 2:**
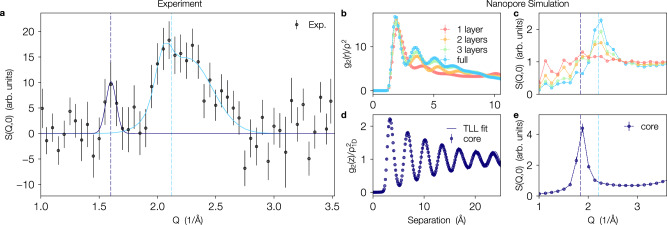
Elastic scattering from ^4^He confined inside MCM-41. **a** Experimental *S*(*Q*, 0) from helium confined to Ar pre-plated pores with an incident wavevector *Q*_in_ of 2.5 Å^−1^ at *T* = 1.6 K. The presented data is for the completely filled pore with (13 mmol g^−1^) with the scattering from the Ar and boundary layer helium (8.68 mmol g^−1^) subtracted (see Supplementary information for the raw data). The shown uncertainties are the statistical errors after the subtraction. The light blue line is a fit of two Gaussians to the scattering centered around 2.1 Å^−1^ originating from the second and third layers of helium in the pores. The purple line is a fit to a Gaussian centered at 1.6 Å^−1^ which we attribute to helium at the pore center. **b**–**e** show the results of quantum Monte Carlo simulations for the structure of ^4^He confined inside a smooth Ar pre-plated nanopore at *T* = 1.6 K. **b** shows the radially averaged density-density correlation function ${g}_{2}(r)=\left\langle \rho (r)\rho (0)\right\rangle$ in units of the density *ρ* = *N*/(*πR*^2^*L*) with *N* the total number of particles inside a pore of length *L* and radius *R* for the four filling fractions highlighted in Fig. 1. **c** The resulting static structure factor for all atoms in the pore. **d** The projected density-density correlations for separations measured along the pore for only those atoms in the central core of the fully filled pore along with a fit to the low energy prediction from Tomonaga-Luttinger liquid theory (solid line). The resulting core-only structure factor in **e** shows a peak at 2*k*_F_ = 2*πρ*_1*D*_ (indicated by a vertical dashed line) where *ρ*_1*D*_ = *N*/*L*. The lighter blue vertical line (also in **a**) corresponds to the separation which minimizes the bulk ^4^He–^4^He interaction potential that controls the structure in the strongly adsorbed outer layers.

Experimental results for elastic scattering from helium confined inside Ar pre-plated MCM-41 are shown in Fig. 2a, where two clear features are apparent. (1) There is a broad peak at ~2.1 Å^−1^ which we attribute to the second and third strongly adsorbed layers of helium in the pores. We note that due to the existence of the adsorption potential, these layers are more dense than helium in the bulk, and their inter-atomic spacing is consistent with this peak as observed in quantum Monte Carlo simulations (panels b, c) which show the predicted pair correlation function ${g}_{2}(r)=\left\langle \rho (r)\rho (0)\right\rangle$ where *ρ*(*r*) is the density of ^4^He and resulting elastic scattering *S*(*Q*, 0) (see Methods for details). Here, different curves correspond to the different filling fractions presented in Fig. 1 and the qualitative agreement between the simulation and experiments supports this picture. (2) A narrow feature at ~1.6 Å^−1^ that we attribute to the atoms at the center of the pore—the core liquid. This peak has been fit to a Gaussian centered at 1.60 ± 0.02 Å^−1^ which corresponds to an atomic spacing of 3.92 ± 0.05 Å. This is in agreement with simulation results for only those atoms in the 1D central core shown in panels d, e where weakly decaying oscillations in the density-density correlations along the pore produce a strong peak in the elastic scattering. Such a peak is predicted by the TLL theory to occur at a wavevector of 2*k*_F_ = 2*πρ*_1*D*_ due to the existence of algebraically decaying Bragg peaks in the structure factor^21^ and has been previously observed in numerical simulations of strictly one dimensional ^4^He^22^. The emergence of a *Fermi* wavevector is a consequence of 1D where the hard core of the ^4^He–^4^He interaction potential dominates making the bosonic system exhibit properties of an ideal gas of spinless fermions. This is consistent with the interpretation that in interacting gapless quantum systems *k*_F_ corresponds to the smallest momentum at which energy can be absorbed.

Thus we can separate contributions of adsorbed layers and central core atoms, which are highlighted by two vertical dashed lines in panels c and e, and repeated in panel a, supporting the interpretation of the experimental results as demonstrating the existence of a one dimensional quasi-liquid of helium with a linear density near *ρ*_1*D*_ ≈ 0.25 Å^−1^. Further evidence comes from the relative intensity of this peak and that due to the first and second layers, which is in agreement with the theoretical number of atoms in the core liquid versus shell region (see Supplementary information).

### Excitations of low-dimensional helium

**Fig. 3:**
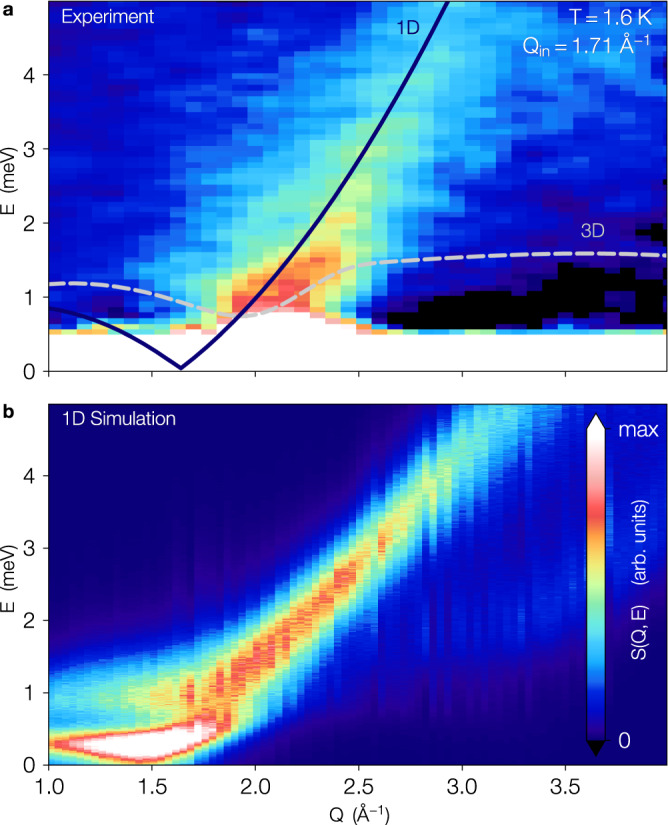
Inelastic scattering of a 1D quantum liquid. **a** The dynamic structure factor, *S*(*Q*, *E*), from helium confined in the Ar plated pores of MCM-41. The scattering was measured at a filling of 13 mmol g^−1^, corresponding to full pores, and the background from the Ar/MCM-41 matrix has been removed with the elastic scattering suppressed. The most prominent feature is the strong excitation that begins at ~1.6 Å^−1^ at *E* = 0 and extends to high energy. This is quite distinct from the scattering that would be expected from either bulk ^4^He or that of ^4^He confined in larger pores where the scattering would be described by the phonon-maxon-roton excitation curve (gray dashed line). For the bulk liquid, the scattering would be most intense around the roton minimum at *Q*_*r*_ ≃ 1.9 Å^−1^ and would plateau at twice the roton energy (~1.5 meV). The purple line shows a theoretical prediction for the threshold energy of a purely 1D quantum liquid of hard spheres with emergent Fermi wavevector 2*k*_F_ = 1.6 Å^−1^ and dimensionless Tomonaga-Luttinger liquid parameter *K* ≃ 1.2. The value for *k*_F_ has been obtained from *S*(*Q*, 0), while *K* comes from fitting the inelastic branch of *S*(*Q*, *E*) (see Fig. 4). **b** The dynamic structure factor of a purely 1D system of ^4^He at *T* = 1.6 K obtained via numerical analytic continuation of the imaginary time scattering function measured via quantum Monte Carlo for a system with *L* = 200 Å. The density was chosen to produce a value of *K* similar to that seen in the experiment.

**Fig. 4:**
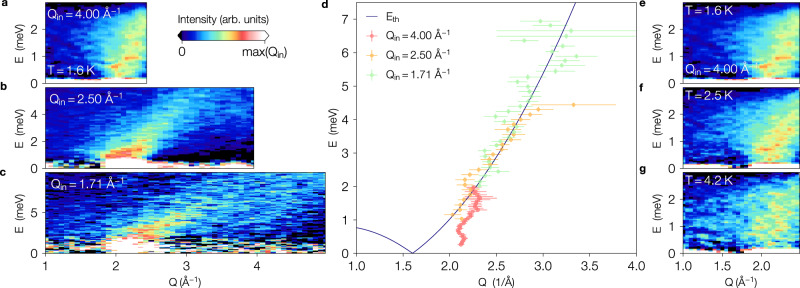
Energy and temperature dependence of the inelastic scattering branch. Left panels: Inelastic scattering from confined ^4^He at various incident wave vectors covering different *Q* − *E* ranges with different resolutions. The plots correspond to incident wavevectors of: **a** 4.00 Å^−1^, **b** 2.50 Å^−1^, and **c** 1.71 Å^−1^. The *Q* scales are the same for all three values, but the energy ranges are different. **d** The peak excitation energies obtained by fitting a Gaussian in energy to the inelastic scattering in **a**–**c** with the *Q* and *E* uncertainties determined in the fit shown as standard error bars. The solid line is the best fit to the threshold energy of a purely 1D quantum liquid of hard spheres. The fit was obtained by fixing *k*_F_ = 0.8 Å^−1^, as extracted from the *S*(*Q*, 0) measurement, and yielded $K=1.1{8}_{-0.20}^{+0.38}$. Right Panels: Inelastic scattering from confined helium at various temperatures above and below the bulk superfluid transition. The measurements have fixed *Q*_in_ = 4.00 Å^−1^ and temperatures of: **e** 1.6 K, **f** 2.5 K, and **g** 4.2 K. The scattering intensity decreases slightly with increasing temperature but remains large even at the highest temperature studied.

We now turn to probing the dynamics of the confined helium within the nanopores. Figures 3a and 4 show the measured inelastic scattering as captured by the dynamic structure factor *S*(*Q*, *E*) while Fig. 3b shows a quantum Monte Carlo prediction for a purely 1D model of ^4^He using differential evolution to analytically continue imaginary time correlations to real frequencies^23^. As described above, the observed *S*(*Q*, *E*) arises primarily for the core quasi-liquid, with the immobile solid layers contributing very little to the inelastic scattering. Figure 3a demonstrates a single well defined inelastic feature which begins at ~1.6 Å^−1^ and its energy increases monotonically and smoothly with increasing wavevector *Q*. We note that this is qualitatively distinct from bulk liquid helium (dashed line) which exhibits a well defined excitation spectrum with a linear phonon mode at low *Q* (*E*_phonon_ ∝ *Q*) and a very intense roton mode with gap Δ at intermediate *Q* = *Q*_*r*_ ≈ 1.9 Å^−1^ (${E}_{{{{{{{{\rm{roton}}}}}}}}}\approx {{\Delta }}+C{(Q-{Q}_{r})}^{2}$) and a plateau at ~ 1.5 meV at *Q* > *Q*_*r*_ due to two roton processes.

While at low-*Q*, the harmonic Tomonaga-Luttinger liquid theory describes a phonon branch where the only excitations are density waves propagating with velocity *v* such that *S*(*Q*, *E*) ≃ *Qδ*(*E* − *ℏvQ*)^24^, at higher *Q*, the dynamic structure factor is known to be markedly different. Here, the effects of band curvature introduce edge singularities in *S*(*Q*, *E*)^25–27^ where low-energy excitations can only proliferate above an energy threshold *E*_th_. By interpreting the excitations of ^4^He confined in Ar-preplated MCM-41 in terms of an effective quantum impurity model for a hole propagating in an otherwise linear TLL^25–28^, the dynamical structure factor can be shown to develop a *Q*-dependent power law singularity at low energies:

1 $$S(Q,E)\propto {{\Theta }}(E-{E}_{{{{{{{{\rm{th}}}}}}}}}(Q))| E-{E}_{{{{{{{{\rm{th}}}}}}}}}(Q){| }^{-\mu (Q)}$$

where for a model of hard core bosons^17,22^

2 $${E}_{{{{{{{{\rm{th}}}}}}}}}(Q)\simeq \frac{4{E}_{{{{{{{{\rm{F}}}}}}}}}}{K}\left[\frac{Q}{2{k}_{{{{{{{{\rm{F}}}}}}}}}}-{\left(\frac{Q}{2{k}_{{{{{{{{\rm{F}}}}}}}}}}\right)}^{2}\right]$$

with *K* the Luttinger parameter and the appearance of the Fermi energy ${E}_{{{{{{{{\rm{F}}}}}}}}}={\hslash }^{2}{k}_{{{{{{{{\rm{F}}}}}}}}}^{2}/(2m)$ further supporting the emergent fermionization of the 1D ^4^He. Here the exponent *μ*(*Q*) is both non-universal (depending on the details of the microscopic interactions and cutoff) as well as momentum dependent, and can vary significantly near 2*k*_F_, even changing sign^17,22^! We interpret the observed inelastic branch in Fig. 3 as corresponding to *E*_th_ and fit the maxima to Eq. (2) over a broad range of *Q* (see Fig. 4a–d) with the value of 2*k*_F_ fixed at 1.6 Å^−1^ as determined by the elastic scattering. This allows us to extract a best fit value of the Luttinger parameter of $K=1.1{8}_{-0.20}^{+0.38}$ which is consistent with microscopic predictions for ^4^He inside smooth nanopores from quantum Monte Carlo^15^ as well as a model of hardcore bosons where $K={(1-{\rho }_{1D}{a}_{1D})}^{2}$ with *a*_1*D*_ the effective 1D scattering length^29^.

The emergence of a 1D quasi-liquid is further supported by Fig. 4e–g which shows the weak temperature dependence of the inelastic scattering below *T* = 4.2 K where the main dispersing feature becomes more diffuse as the temperature is increased. This is consistent with the extracted value of *K* ≃ 1.2 which sets the relevant scale below which we expect to observe TLL behavior to be *T* ≪ *ℏvQ*/*k*_B_ ≃ 4*E*_F_/(*Kk*_B_) ≃ 13 K for *Q* ≃ 2*k*_F_ where *k*_B_ is Boltzmann’s constant. Thus the helium core liquid still retains its 1D excitation spectrum even above the bulk superfluid temperature.

## Discussion

We have created a nano-engineered confinement environment for helium that has allowed us to observe 1D quantum liquid behavior beyond the Luttinger liquid paradigm and obtained information on the microscopic structure inside the pores via quantum Monte Carlo simulations. The next steps are many, including exploring the ability to control the filling fraction via pressure near the full pore regime to tune the density (and thus Luttinger parameter) of the confined liquid, manifest as a modified slope of the inelastic threshold *E*_th_ in the excitation spectrum. Replacing bosonic ^4^He with the fermionic isotope ^3^He is even more exciting and would potentially open the door for the observation of spin-mass separation, where spin and density waves propagate with different excitation velocities.

## Methods

### Sample characterization

MCM-41 is a mesoporous material with a hierarchical structure consisting of cylindrical pores arranged in a hexagonal lattice. It is produced using a surfactant templating technique that produces pores that are monodisperse, unidirectional, and have a regular 2D hexagonal structure. The typical aspect ratio of the pores is ~1000:1 making them an attractive medium for studies of 1D behavior. The sample and characterization techniques have been reported previously and we will briefly review them here.

Our sample was obtained from Sigma-Aldrich^30^ and was characterized using X-ray powder diffraction and N_2_ gas adsorption isotherm measurements^10^. The X-ray diffraction data indicated that the sample consisted of a single phase with pores arranged on a hexagonal lattice with a lattice constant of 4.7 nm. A Brunauer-Emmett-Teller (BET) analysis^31^ of the N_2_ isotherm gave a surface area of 915 m^2^/g. The pore diameter size distribution was calculated using the Kruk-Jaroniec-Sayari method^32^ and was found to be Gaussian with a mean value of 3.0 nm and a full-width at half-maximum of 0.3 nm.

Adsorption isotherms were also carried out with research grade Ar gas at 90 K to determine the monolayer coverage^18^. A BET analysis of the isotherm yielded a monolayer coverage of 8.994 mmol g^−1^. This monolayer coverage, when combined with the measured surface area, yields an aerial coverage of 0.59 Å^−2^ and, using the van der Waals radius for Ar, a monolayer density of *n*_Ar_ = 0.017 Å^−3^.

We also carried out ^4^He isotherms on MCM-41 preplated with a single monolayer of Ar. The Ar pre-plating was carried out at 90 K and then the sample was slowly cooled to 4.2 K over the course of several hours. ^4^He isotherms were then carried out at 4.2 K using standard volumetric techniques. The results are shown in Fig. 1 of the main text. The initially adsorbed ^4^He is strongly bound to the surface of the MCM-41 resulting in zero pressure rise until ~7.5 mmol g^−1^ has been adsorbed. There is a small region between ~7.5 nmol/g^−1^ and 13 mmol g^−1^ where the pressure increases. Once a filling of 13 mmol g^−1^ has been reached no additional helium is adsorbed into the pores until the pressure is close to the bulk vapor pressure *P*_0_. Once *P*/*P*_0_ is greater than ~0.9, ^4^He capillary condenses between the MCM-41 grains.

### Neutron scattering

Neutron scattering studies of ^4^He in Ar preplated MCM-41 were carried out using the Disc Chopper Spectrometer (DCS) at the NIST Center for Neutron Research^33^. This instrument is a direct geometry time-of-flight chopper spectrometer which views a cold moderator. High speed choppers are used to create a pulsed neutron beam with a well defined incident wavelength. Neutrons scattered by the sample are detected by a secondary spectrometer consisting of 913 ^3^He detectors 4.01 m from the sample at scattering angles from 5^∘^ to 140^∘^. Standard data reduction routines^34^ are used to convert the observed scattering to the dynamic structure factor *S*(*Q*, *E*).

The sample for these studies consisted of 6.13 g of MCM-41 inside a cylindrical aluminum can. The MCM-41 was in the form of cylindrical pellets 1.25 cm in diameter and 1 cm high with a mass of 0.875 g. The pellets were baked in vacuum at 120 ^∘^C to remove adsorbed water vapor. The sample was then transferred to an aluminum sample cell in a nitrogen glove box. The cell was a cylindrical aluminum can of outer diameter 1.5 cm, a height of 6 cm, and a wall thickness of 1 mm. The pellets were separated by cadmium spacers to reduce multiple scattering. A top-loading liquid helium cryostat with aluminum tails, commonly referred to as an “orange" cryostat, was used to obtain the low temperatures examined in this study. A silicon diode was used to monitor the sample temperature.

Incident wavevectors of 4.0, 2.5 and 1.71 Å^−1^ were used (see Fig. 4 in the main text for incident wavevector dependence). Longer incident wavelengths offer the advantage of better energy resolution but at significantly decreased intensity and range of momentum transfers, *Q*, accessible. The studies at 4.0 Å^−1^ had an energy resolution of 93.6 *μ*eV. However, the flux was limited to 1.05 × 10^5^ neutrons/cm^2^/s and the maximum *Q* was 2.9 Å^−1^. Due to this lower flux and limited Q range we limited these studies to two fillings of monolayer and slightly overfilled pores. More extensive studies at a variety of fillings were carried out at 2.5 Å^−1^. The energy resolution was significantly larger (772 *μ*eV) but with much larger incident flux (8.75 × 10^5^ neutrons/cm^2^/s) and Q range (4.6 Å^−1^). A limited number of measurements were also carried out at 1.71 Å^−1^. These measurements had significantly worse energy resolution (2370 *μ*eV) but a much expanded Q range (6.8 Å^−1^). However, since DCS is located on a cold moderator the flux is significantly decreased (1.89 × 10^5^ neutrons/cm^2^/s) at these short wavelengths.

### Theoretical modeling

An ab initio model of superfluid ^4^He confined inside ordered nanoporous MCM-41 pre-pated with argon gas can be constructed from the superposition of single pores, each described by a *N*-body Hamiltonian:

3 $$H=-\frac{{\hslash }^{2}}{2m}\mathop{\sum }\limits_{i = 1}^{N}{\nabla }_{i}^{2}+\mathop{\sum }\limits_{i = 1}^{N}{U}_{{{{{{{{\rm{pore}}}}}}}}}({{{{{{{{\bf{r}}}}}}}}}_{i})+\frac{1}{2}\mathop{\sum }\limits _{i,j}{V}_{{{{{{{{\rm{He}}}}}}}}}({{{{{{{{\bf{r}}}}}}}}}_{i}-{{{{{{{{\bf{r}}}}}}}}}_{j})$$

where *m* is the mass of a single ^4^He atom located at position **r**_*i*_ = (*x*_*i*_, *y*_*i*_, *z*_*i*_) confined inside a nanopore by ${U}_{{{{{{{{\rm{pore}}}}}}}}}$ and interacting with other He atoms through *V*_He_. Both potential energy terms arise from induced dipole-dipole interactions. ${U}_{{{{{{{{\rm{pore}}}}}}}}}$ was recently determined for the specific system under consideration here^18^ while *V*_He_ is known to high precision^35–37^.

#### Quantum Monte Carlo Method

A system of confined helium described by Eq. (3) was simulated using a quantum Monte Carlo algorithm exploiting path integrals^18,38,39^. *T* > 0 expectation values of observables ${{{{{{{\mathcal{O}}}}}}}}$ were sampled via

4 $$\left\langle {{{{{{{\mathcal{O}}}}}}}}\right\rangle =\frac{1}{{{{{{{{\mathcal{Z}}}}}}}}}{{{{{{{\rm{Tr}}}}}}}}\left\{{{{{{{{\mathcal{O}}}}}}}}\,{{{{{{{{\rm{e}}}}}}}}}^{-\beta H}\right\}$$

where *β* = 1/*k*_B_*T* is the inverse temperature and the partition function ${{{{{{{\mathcal{Z}}}}}}}}={{{{{{{\rm{Tr}}}}}}}}{{{{{{{{\rm{e}}}}}}}}}^{-\beta H}$ can be written as a sum of discrete imaginary time paths (worldlines) over the set of all permutations ${{{{{{{\mathcal{P}}}}}}}}$ of the first quantized labels of the *N* indistinguishable ^4^He atoms:

5 $${{{{{{{\mathcal{Z}}}}}}}}\simeq \frac{1}{{(4{{\Lambda }}\tau )}^{3NM/2}}\frac{1}{N!} \mathop{\sum }\limits_{{{{{{{{\mathcal{P}}}}}}}}}\left[\mathop{\prod }\limits_{\alpha = 0}^{M-1}\int {{{{{{{\mathcal{D}}}}}}}}{R}_{\alpha }\right]{{{{{{{{\rm{e}}}}}}}}}^{-\mathop{\sum }\nolimits_{\alpha = 0}^{M-1}{{{{{{{{\mathcal{S}}}}}}}}}_{\alpha }}\,.$$

Here *τ* = *β*/*M* is the imaginary time step where $M\in {\mathbb{Z}}\gg 1$ and *R*_*α*_ ≡ ***r***_*α*,1_, ***r***_*α*,2_, …, ***r***_*α*,*N*_ are the spatial positions of the particles at imaginary time slice *α*. Bosonic symmetry restricts ${{{{{{{\mathcal{P}}}}}}}}{R}_{M}={R}_{0}$ and we have employed the short-hand notation $\int {{{{{{{\mathcal{D}}}}}}}}{R}_{\alpha }\equiv \mathop{\prod }\nolimits_{i = 1}^{N}\int {{{{{{{\rm{d}}}}}}}}{{{{{{{{\boldsymbol{r}}}}}}}}}_{\alpha ,i}$ and Λ = *ℏ*^2^/(2*m*). Discrete imaginary time-step errors are suppressed to O(*τ*^4^)^40^ through an effective imaginary time action

6 $${{{{{{{{\mathcal{S}}}}}}}}}_{\alpha } ={\,}	\mathop{\sum }\limits_{i = 1}^{N}\frac{| | {{{{{{{{\bf{r}}}}}}}}}_{\alpha ,i}-{{{{{{{{\bf{r}}}}}}}}}_{\alpha +1,i}| {| }^{2}}{4{{\Lambda }}\tau }+\,\tau [1+\frac{1}{3}{(-1)}^{\alpha }]{{{{{{{\mathcal{V}}}}}}}}({R}_{\alpha })\\ 	+\ {\tau }^{3}[1-{(-1)}^{\alpha }]\frac{{{\Lambda }}}{9}\mathop{\sum }\limits_{i = 1}^{N}| | {\nabla }_{i}{{{{{{{\mathcal{V}}}}}}}}({R}_{\alpha })| {| }^{2}$$

where

7 $${{{{{{{\mathcal{V}}}}}}}}({R}_{\alpha })\equiv \mathop{\sum }\limits_{i = 1}^{N}{U}_{{{{{{{{\rm{pore}}}}}}}}}({{{{{{{{\boldsymbol{r}}}}}}}}}_{\alpha ,i})+\mathop{\sum }\limits_{i\,{ < }\,j}{V}_{{{{{{{{\rm{He}}}}}}}}}({{{{{{{{\boldsymbol{r}}}}}}}}}_{\alpha ,i}-{{{{{{{{\boldsymbol{r}}}}}}}}}_{\alpha ,j})\,.$$

Simulations were performed at *T* = 1.6 K for four chemical potentials: *μ*/*k*_B_ = −47 K, −27 K, −19 K and −7 K that were identified as representative of the nanopores at different stages of filling– from a single adsorbed layer at *μ*/*k*_B_ = −47 K to a fully filled pore at *μ*/*k*_B_ = −7 K (see Fig. 1 in the main text). The pores had outer radius *R* = 15.51 Å and length *L* = 50 Å and all simulations were performed in the grand canonical ensemble yielding *N* ≈ 600 helium atoms for *μ*/*k*_B_ = −7 K. Trotter errors were deemed to be smaller than statistical uncertainties for *τ* ⋅ *k*_B_ = 1/250 K^−1^, which was used for all simulations. Further details (including the effects of the finite pore length) are reported in ref. ^18^. The quantum Monte Carlo software used to produce all results is available online^41^.

#### Observables

Using the quantum Monte Carlo method described above we measure the radial density (Fig. 1):

8 $${\rho }_{{{{{{{{\rm{rad}}}}}}}}}(r)=\left\langle \mathop{\sum }\limits_{i = 1}^{N}\delta \left(\sqrt{{x}_{i}^{2}+{y}_{i}^{2}}-r\right)\right\rangle \,,$$

the density-density correlation function (Fig. 2b,d):

9 $${g}_{2}(r)=\left\langle \rho (r)\rho (0)\right\rangle =\left\langle \frac{V}{{N}^{2}}\mathop{\sum }\limits_{i\ne j}\delta (r-| {{{{{{{{\boldsymbol{r}}}}}}}}}_{i}-{{{{{{{{\boldsymbol{r}}}}}}}}}_{j}| )\right\rangle \,,$$

and the structure factor (elastic scattering, Fig. 2c, e):

10 $$S({{{{{{{\boldsymbol{Q}}}}}}}})=\left\langle \frac{1}{N}\rho ({{{{{{{\boldsymbol{Q}}}}}}}})\rho (-{{{{{{{\boldsymbol{Q}}}}}}}})\right\rangle .$$

Here *V* is the accessible volume of the pore and $\rho ({{{{{{{\boldsymbol{Q}}}}}}}})=\mathop{\sum }\nolimits_{i = 1}^{N}\exp (-i{{{{{{{\boldsymbol{Q}}}}}}}}\cdot {{{{{{{{\boldsymbol{r}}}}}}}}}_{i})$. The core versions of these estimators in Fig. 2d, e include only those ^4^He atoms with $\sqrt{{x}_{i}^{2}+{y}_{i}^{2}} \, < \, 1.72$ Å identified as the location of the first minimum of *ρ*_rad_(*r*) shown in Fig. 1b for the full pore with *μ*/*k*_B_ = −7 K.

For the inelastic scattering *S*(*Q*, *E*) in Fig. 3b, simulations were performed on a purely one-dimensional system of ^4^He with *L* = 200 Å in the canonical ensemble at fixed *ρ*_1*D*_ = 0.14 Å^−1^ and *T* = 1.6 K. The intermediate scattering function:

11 $$F({{{{{{{\boldsymbol{Q}}}}}}}},\alpha \tau )=\frac{1}{N}\left\langle \mathop{\sum }\limits_{j,k}{e}^{-i{{{{{{{\boldsymbol{Q}}}}}}}}\cdot {{{{{{{{\boldsymbol{r}}}}}}}}}_{\alpha ,j}}{e}^{i{{{{{{{\boldsymbol{Q}}}}}}}}\cdot {{{{{{{{\boldsymbol{r}}}}}}}}}_{0,k}}\right\rangle$$

is related to the dynamic structure factor *S*(***Q***, *E*) via:

12 $$F({{{{{{{\boldsymbol{Q}}}}}}}},\tau )=\frac{1}{\hslash }\int\nolimits_{0}^{\infty }S({{{{{{{\boldsymbol{Q}}}}}}}},E)\left[{e}^{-\tau E}+{e}^{-(\beta -\tau )E}\right]{{{{{{{\rm{d}}}}}}}}E$$

which can be inverted using a recently introduced parameter-free differential evolution algorithm for imaginary time correlation functions^23^.

### Tomonaga-Luttinger liquid predictions

The one-dimensional density-density correlation function can be computed within the effective quantum hydrodynamic theory to be^15,42^:

13 $$\langle \rho (z)\rho (0)\rangle ={\,}	{\rho }_{1D}^{2}+\frac{K}{2{\pi }^{2}}\frac{{d}^{2}}{d{z}^{2}}\ln {\theta }_{1}\left[\frac{\pi z}{L},{e}^{-\pi \hslash v/L{k}_{{{{{{{{\rm{B}}}}}}}}}T}\right]\\ 	+{{{{{{{\mathcal{A}}}}}}}}\cos \left(2\pi {\rho }_{1D}z\right){\left\{\frac{2\eta \left(\frac{i\hslash v}{L{k}_{{{{{{{{\rm{B}}}}}}}}}T}\right){e}^{-\pi \hslash v/6L{k}_{{{{{{{{\rm{B}}}}}}}}}T}}{{\theta }_{1}\left(\frac{\pi z}{L},{e}^{-\pi \hslash v/L{k}_{{{{{{{{\rm{B}}}}}}}}}T}\right)}\right\}}^{2K}$$

where *η*( ⋅ ) is the Dedekind eta function and *θ*_1_(*z*, *q*) is the Jacobi theta function of the first kind. This expression depends on 3 parameters: *K*, *v* and ${{{{{{{\mathcal{A}}}}}}}}$ with *ρ*_1*D*_ being determined from simulations as *ρ*_1*D*_ = *N*/*L*. In practice it is beneficial to first fit the envelope of decay from the asymptotic form:

14 $$\langle\rho(z) \rho(0)\rangle {\mathop{\approx}\limits^{L\to\infty}_{T\to 0}} \rho_{0}^{2}-\frac{K}{2 \pi^{2} z^{2}}+\frac{{{{{\mathcal{A}}}}}}{z^{2K}} \cos\left(2 \pi \rho_{0} z\right)$$

to obtain reasonable initial values for the parameters before performing a full non-linear fit to Eq. (13). For the core ^4^He atoms with *ρ*_1*D*_ = 0.293(2) shown in Fig. 2d we find *K* = 0.15(4), *ℏv*/*k*_*B*_ = 8(3)Å K and ${{{{{{{\mathcal{A}}}}}}}}=0.036(2)$. The different value of *K* extracted in this simulation compared to that found by analyzing the experimentally determined *S*(*Q*, *E*) is due to this pore being closer to the fully filled regime with *ρ*_1*D*_ ≈ 0.3 as opposed to *ρ*_1*D*_ ≈ 0.25 found in the experiment.

## Supplementary information

Supplementary Information

## Data Availability

The experimental and processed quantum Monte Carlo data generated in this study have been deposited in a github repository under accession code 10.5281/zenodo.6112399^43^. The complete (raw) simulation data set has been deposited on Zenodo under accession code 10.5281/zenodo.6012498^44^.

## Code availability

The code and scripts used to process data and generate all figures in this paper are available online at https://github.com/DelMaestroGroup/papers-code-preplated-nanopores-scattering^43^.

## References

[1] Bishop DJ, Reppy JD (1978). Study of the Superfluid Transition in Two-DimensionalHe4Films. Phys. Rev. Lett..

[2] Godfrin H, Beauvois K, Sultan A, Krotscheck E, Dawidowski J, Fåk B, Ollivier J (2021). Dispersion relation of Landau elementary excitations and thermodynamic properties of superfluid He4. Phys. Rev. B.

[3] Tomonaga SI (1950). Remarks on Bloch’s Method of Sound Waves applied to Many-Fermion Problems. Prog. Theor. Phys..

[4] Luttinger JM (1963). An Exactly Soluble Model of a Many-Fermion System. J. Math. Phys..

[5] Mattis DC, Lieb EH (1965). Exact Solution of a Many-Fermion System and Its Associated Boson Field. J. Math. Phys..

[6] Haldane FDM (1981). Effective Harmonic-Fluid Approach to Low-Energy Properties of One-Dimensional Quantum Fluids. Phys. Rev. Lett..

[7] Duc P-F, Savard M, Petrescu M, Rosenow B, Maestro AD, Gervais G (2015). Critical flow and dissipation in a quasi–one-dimensional superfluid. Sci. Adv..

[8] Botimer J, Taborek P (2016). Pressure driven flow of superfluid ^4^He through a nanopipe. Phys. Rev. Fluids.

[9] Wada N, Taniguchi J, Ikegami H, Inagaki S, Fukushima Y (2001). Helium-4 Bose Fluids Formed in One-Dimensional 18 Å Diameter Pores. Phys. Rev. Lett..

[10] Prisk TR, Das NC, Diallo SO, Ehlers G, Podlesnyak AA, Wada N, Inagaki S, Sokol PE (2013). Phases of superfluid helium in smooth cylindrical pores. Phys. Rev. B.

[11] Bossy J, Ollivier J, Glyde HR (2019). Phonons, rotons, and localized Bose-Einstein condensation in liquid ^4^He confined in nanoporous FSM-16. Phys. Rev. B.

[12] Vekhov Y, Hallock RB (2012). Mass Flux Characteristics in Solid ^4^He for *T* > 100 mK: Evidence for Bosonic Luttinger-Liquid Behavior. Phys. Rev. Lett..

[13] Gordillo M, Boronat J, Casulleras J (2000). Quasi-one-dimensional 4He inside carbon nanotubes. Phys. Rev. B.

[14] Boninsegni M, Kuklov AB, Pollet L, Prokof’ev NV, Svistunov BV, Troyer M (2007). Luttinger Liquid in the Core of a Screw Dislocation in Helium-4. Phys. Rev. Lett..

[15] Del Maestro A, Boninsegni M, Affleck I (2011). ^4^He Luttinger Liquid in Nanopores. Phys. Rev. Lett..

[16] Markić LV, Glyde HR (2015). Superfluidity, BEC, and dimensions of liquidHe4in nanopores. Phys. Rev. B.

[17] Bertaina G, Motta M, Rossi M, Vitali E, Galli DE (2016). One-Dimensional Liquid 4He: Dynamical Properties beyond Luttinger-Liquid Theory. Phys. Rev. Lett..

[18] Nichols NS, Prisk TR, Warren G, Sokol P, Del Maestro A (2020). Dimensional reduction of helium-4 inside argon-plated MCM-41 nanopores. Phys. Rev. B.

[19] Nava A, Giuliano D, Nguyen PH, Boninsegni M (2022). Quasi-one-dimensional ^4^He in nanopores. Phys. Rev. B.

[20] Bryan MS, Prisk TR, Sherline TE, Diallo SO, Sokol PE (2017). Bulklike excitations in nanoconfined liquid helium. Phys. Rev. B.

[21] Luther A, Peschel I (1974). Single-particle states, Kohn anomaly, and pairing fluctuations in one dimension. Phys. Rev. B.

[22] Motta M, Vitali E, Rossi M, Galli DE, Bertaina G (2016). Dynamical structure factor of one-dimensional hard rods. Phys. Rev. A.

[23] Nichols, N. S., Sokol, P., & Del Maestro, A. A parameter-free differential evolution algorithm for the analytic continuation of imaginary time correlation functions. arXiv:2201.04155 (2022). 10.1103/PhysRevE.106.025312 36109945

[24] Dzyaloshinskii IE, Larkin AI (1973). Correlation functions for a one-dimensional Fermi system with long-range interaction (Tomonaga model). Zh. Eksp. Teor. Fiz..

[25] Pustilnik M, Khodas M, Kamenev A, Glazman LI (2006). Dynamic Response of One-Dimensional Interacting Fermions. Phys. Rev. Lett..

[26] Pereira RG, White SR, Affleck I (2008). Exact Edge Singularities and Dynamical Correlations in Spin-1/2 Chains. Phys. Rev. Lett..

[27] Imambekov A, Schmidt TL, Glazman LI (2012). One-dimensional quantum liquids: Beyond the Luttinger liquid paradigm. Rev. Mod. Phys..

[28] Imambekov A, Glazman LI (2009). Phenomenology of One-Dimensional Quantum Liquids Beyond the Low-Energy Limit. Phys. Rev. Lett..

[29] Olshanii M (1998). Atomic Scattering in the Presence of an External Confinement and a Gas of Impenetrable Bosons. Phys. Rev. Lett..

[30] Sigma-Aldrich (2008). Synthesis of Mesoporous Materials. Mater. Matters.

[31] Brunauer S, Emmett PH, Teller E (1938). Adsorption of Gases in Multimolecular Layers. J. Am. Chem. Soc..

[32] Jaroniec M, Kruk M, Olivier JP (1999). Standard Nitrogen Adsorption Data for Characterization of Nanoporous Silicas. Langmuir.

[33] Copley J, Cook J (2003). The Disk Chopper Spectrometer at NIST: a new instrument for quasielastic neutron scattering studies. Chem. Phys..

[34] Azuah RT, Kneller LR, Qiu Y, Tregenna-Piggott PLW, Brown CM, Copley JRD, Dimeo RM (2009). DAVE: A comprehensive Software Suite for the Reduction, Visualization, and Analysis of Low Energy Neutron Spectroscopic Data. J. Res. Natl. Inst. Stand. Technol..

[35] Aziz RA, Nain VPS, Carley JS, Taylor WL, McConville GT (1979). An accurate intermolecular potential for helium. J. Chem. Phys..

[36] Przybytek M, Cencek W, Komasa J, Łach G, Jeziorski B, Szalewicz K (2010). Relativistic and Quantum Electrodynamics Effects in the Helium Pair Potential. Phys. Rev. Lett..

[37] Cencek W, Przybytek M, Komasa J, Mehl JB, Jeziorski B, Szalewicz K (2012). Effects of adiabatic, relativistic, and quantum electrodynamics interactions on the pair potential and thermophysical properties of helium. J. Chem. Phys..

[38] Ceperley DM (1995). Path integrals in the theory of condensed helium. Rev. Mod. Phys..

[39] Boninsegni M, Prokof’ev N, Svistunov B (2006). Worm Algorithm for Continuous-Space Path Integral Monte Carlo Simulations. Phys. Rev. Lett..

[40] Suzuki M (1990). Fractal decomposition of exponential operators with applications to many-body theories and monte carlo simulations. Phys. Lett. A.

[41] Del Maestro, A. Path Integral Quantum Monte Carlo. Online (2022) https://code.delmaestro.org.

[42] Del Maestro A (2012). A Luttinger Liquid Core Inside Helium-4 Filled Nanopores. Int. J. Mod. Phys. B.

[43] Del Maestro, A., Nichols, N. S., & Sokol, P. E. Github Repository: DelMaestroGroup/papers-code-preplated-nanopores-scattering. 10.5281/zenodo.6112399 (2022).

[44] Del Maestro A., Nichols, N. S. Quantum Monte Carlo data for 4He inside Ar-Preplated MCM-41 Nanopores. 10.5281/zenodo.6012499 (2022).

