# Supplementary material for: Experimental realization of one dimensional helium
Source: Nat Commun. 2022 Jun 7;13:3168. doi: 10.1038/s41467-022-30752-3 (PMC9174257; doi:10.1038/s41467-022-30752-3)
Supplement: Supplementary file 1 — Supplementary Information [file 41467_2022_30752_MOESM1_ESM.pdf]

Supplementary Information for "Experimental Realization of One Dimensional Helium"

Adrian Del Maestro, Nathan S. Nichols, Timothy R. Prisk, Garfield Warren, Paul E. Sokol

ELASTIC SCATTERING

Experimental adsorption isotherms were used to connect the experimental results to quantum Monte Carlo calculations of the layer density in the pores [1]. As noted in the main text, pore completion occurs at 13 mmol/g and this value was used to normalize to the results of Nichols *et al.* [1] to provide filling ranges for each of the layers. These computed values are provided in Table 1.

Layer	Filling (mmol/g)	Fraction of atoms	Spacing (Å)
Layer 1	0.00 - 6.53	0.50	3.16
Layer 2	6.53 - 10.73	0.32	3.24
Layer 3	10.73 - 12.71	0.15	3.53
Pore Center	12.73 - 13.01	0.02	3.9

Supplementary Table 1. **Layer Filling Properties.** The range of fillings (in mmol/g), fraction of atoms, and inter-layer spacing for each of the layers. The filling scale is obtained by scaling the full pore filling, as determined from adsorption isotherms, to quantum Monte Carlo calculations.

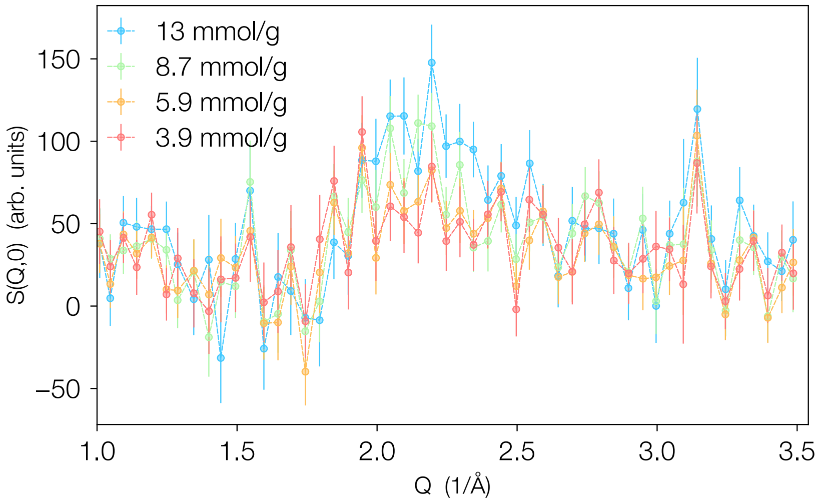

Supplementary Figure 1. **Elastic scattering from confined helium.** Experimental measurements of $S(Q,0)$ at fillings of 3.89 (red), 5.91 (orange), 8.68 (green) and 13.0 (blue) mmol/g corresponding roughly to those filling fractions where layer completion may occur. Measurements were carried out with an incident wavelength of 2.5 Å^{-1} and the scattering from the Ar plated MCM-41 has been subtracted, with the uncertainty shown as standard errors on the data points. Note the agreement with Figure 2**c** in the main text computed from quantum Monte Carlo simulations.

As can be seen in Table 1, half of the atoms reside in the first layer and are strongly bound to the Ar/MCM-41 surface. Layers 2 and 3, which are less strongly bound but still solid like, account for an additional 47% of the material in the pores. Finally, the central core liquid, since it is only 1 atom in lateral extent, accounts for only 2-3% of the helium in the pores.

The elastic scattering with helium fillings of 3.89, 5.91, 8.68, and 13.0 mmol/g is shown in Figure 1. Figure 2**a** in the main text is computed from performing subtractions within this data set and we note the strong similarity with the simulation results presented in Figure 2**c** of the main text. The two lower fillings correspond to a nearly completed monolayer and nearly completed second layer. The scattering is strong at small Q then rapidly decreases in intensity

with increasing Q due to grain size of the MCM-41. There is also a relatively broad peak at $\sim$2.1 Å^{-1} and a relatively constant scattering at higher Q. We attribute this scattering to the solid layers that are forming near the pore wall at larger filling. The peak corresponds to an interatomic spacing of $\sim$3 Å consistent with the values in Table 1. We also note that the increase in intensity of the 8.68 mmol/g results with respect to those for 5.91 mmol/g is consistent with the expectation that the monolayer contains 50% of the atoms in the full pore while the second layer only contributes and additional 30%.

We note that the peak at 1.6 Å^{-1} is present but does not stand out clearly in these plots. One must take into consideration that the core liquid represents only 2% of the total scattering while layers 1-3 represent 98% of the intensity. Also, a remnant of the peak is still visible at 8.68 mmol/g. We believe this is due to the fact that this sequence of measurements was carried out upon desorption of ^{4}He from the pores and that some hysteresis occurred leaving a small amount of core liquid still present.

SUPPLEMENTARY REFERENCES

[1] N. S. Nichols, T. R. Prisk, G. Warren, P. Sokol, and A. Del Maestro, Dimensional reduction of helium-4 inside argon-plated MCM-41 nanopores, Phys. Rev. B **102**, 144505 (2020).
